# Robotic Pharmacy Implementation and Outcomes in Saudi Arabia: A 21-Month Usability Study

**DOI:** 10.2196/28381

**Published:** 2021-09-01

**Authors:** Hisham Momattin, Shokry Arafa, Shahad Momattin, Rayan Rahal, James Waterson

**Affiliations:** 1 Mouwasat Medical Services Dammam Saudi Arabia; 2 King Faisal University, Al Hassa Dammam Saudi Arabia; 3 Medication Management Solutions Becton Dickinson Limited Dubai United Arab Emirates; 4 Medical Affairs Medication Management Solutions Becton Dickinson Limited Dubai United Arab Emirates

**Keywords:** patient satisfaction, automation, integration, medication error, outpatient, medication management, usability, medication dispensing, robotics, pharmacy, medication records, error, record, implementation, outcome

## Abstract

**Background:**

We describe the introduction, use, and evaluation of an automation and integration pharmacy development program in a private facility in Saudi Arabia. The project was specifically undertaken to increase throughput, reduce medication dispensing error rates, improve patient satisfaction, and free up pharmacists’ time to allow for increased face-to-face consultations with patients.

**Objective:**

We forecasted growth of our outpatient service at 25% per annum over 5- and 10-year horizons and set out to prepare our outpatient pharmacy service to meet this demand. Initial project goals were set as a 50% reduction in the average patient wait time, a 15% increase in patient satisfaction regarding pharmacy wait time and pharmacy services, a 25% increase in pharmacist productivity, and zero dispensing errors. This was expected to be achieved within 10 months of go-live. Realignment of pharmacist activity toward counseling and medication review with patients was a secondary goal, along with the rapid development of a reputation in the served community for patient-centered care.

**Methods:**

Preimplementation data for patient wait time for dispensing of prescribed medications as a specific measure of patient satisfaction was gathered as part of wider ongoing data collection in this field. Pharmacist activity and productivity in terms of patient interaction time were gathered. Reported and discovered dispensing errors per 1000 prescriptions were also aggregated. All preimplementation data was gathered over an 11-month period.

**Results:**

From go-live, data were gathered on the above metrics in 1-month increments. At the 10-month point, there had been a 53% reduction in the average wait time, a 20% increase in patient satisfaction regarding pharmacy wait time, with a 22% increase in overall patient satisfaction regarding pharmacy services, and a 33% increase in pharmacist productivity. A zero dispensing error rate was reported.

**Conclusions:**

The robotic pharmacy solution studied was highly effective, but a robust upstream supply chain is vital to ensure stock levels, particularly when automated filling is planned. The automation solution must also be seamlessly and completely integrated into the facility’s software systems for appointments, medication records, and prescription generation in order to garner its full benefits. Overall patient satisfaction with pharmacy services is strongly influenced by wait time and follow-up studies are required to identify how to use this positive effect and make optimal use of *freed-up* pharmacist time. The extra time spent by pharmacists with patients and the opportunity for complete overview of the patient’s medication history, which full integration provides, may allow us to address challenging issues such as medication nonadherence. Reduced wait times may also allow for smaller prescription fill volumes, and more frequent outpatient department visits, allowing patients to have increased contact time with pharmacists.

## Introduction

### Background

An article submitted to the *American Journal of Hospital Pharmacy* in 1967 identified how “outpatient visits are increasing at a rapid rate and administrative adjustments will be needed to manage larger outpatient prescription volumes” [1]. The authors laid out how, “[N]ew methods and procedures must be developed to reduce patient wait time, provide the physician and the pharmacist with information pertinent to drug therapy and increase productivity through the elimination of administrative detail which can be handled better through automation,” and how although “…inpatient pharmacy functions have received a considerable amount of publicity in the literature, little work has been accomplished in this area with regard to outpatient dispensing” [1].

The situation remains similar in 2020. Outpatient visits continue to rise year-on-year, with increasingly complex patients being handled by these departments, and there remains a paucity of literature on the application of automation in outpatients to help handle this increasing workload and to deploy the outpatient pharmacy department’s human resources more effectively. There is also considerable political and financial pressure on health care decision-makers to optimize the utilization of resources and to improve services for patients, while ensuring that any technology that is deployed definitively adds quantifiable health economic value. The size of any initial investment in health technology and automation is inevitably significant and requires substantial decisions to be taken about funding; the need for change; and required re-engineering of a facility’s infrastructure and established hospital and department procedures, policies, and workflows [2].

A reasonable number of studies and meta-analyses related to automation processes for inpatient environments have been conducted; some of these can be extrapolated to the outpatient department but only with the caveat that while the 2 settings share some elements, there are also distinct differences in workflow challenges, safety concerns, service elements, and staffing.

A recent systematic literature review of automated and semiautomated drug distribution systems (DDSes) in acute care hospitals evaluated effectiveness in terms of medication safety, time, and costs of medication management [3]. A general conclusion was that patient safety improved with automation, with a reduction in medication errors in both automated and semiautomated DDS. About 24 studies in the review have explored the impact of DDS in terms of labor time, staffing workload, and changes in work processes; however, only 6 studies have explored the economic outcomes. These studies found that highly centralized systems for dispensing saved more time than decentralized arrangements, and it is also notable that although all the DDSes studied decreased medication errors, many of the systems still incurred prescribing errors. These findings may be attributed to the failure to integrate between prescription and dispensing/administering systems or the reliance upon decentralized systems *knitting together*, rather than ensuring seamless information transfer through a fully integrated system. It is notable that, to assess its ability to reduce administration and dispensing error rates, in a 1-center study of an automatic storage and picking system in a pediatric hospital, a computerized provider order entry (CPOE) system was fully integrated into both the existing manual system for preparing daily unit dose drugs and the automated storage and picking system [4]. The study focused on inpatient unit dosing rather than the dispensing of boxed medications for self-administration, but the metrics of wrong medicine, wrong dosage, and wrong pharmaceutical form can apply equally to inpatient and outpatient dispensing. In this study automation showed an error rate reduction with a risk ratio of 3.52, with wrong medicine and wrong dosage being the most prominent areas of error reduction.

Patients and clinicians are concerned over medication safety but a second priority for patients, particularly outpatients, is the time spent waiting for medications to be dispensed [5]. The most common method of outpatient dispensing is for original-pack medications to be given to the patient rather than unit-dose or blister-packs. This method has advantages for automation, as it requires less fine manipulation of the dispensed medication and allows for a relatively faster throughput and service to the patient [6].

A review of the limited literature focusing directly on outpatient and pharmacy robotics showed the same emphasis on medication safety as with inpatient studies, with an identifiable improvement following automation [7]. Productivity, as measured by prescription filling time, also improved with automation in the reviewed studies with a reduction in the required personnel of between 0.3 and 1.4 full-time employees (FTEs) and increases in items picked per FTE per hour. The review found, however, that despite the decrease in both patients’ wait times and prescription filling time, there was no observable change in staff perception of workload.

With regard to original pack dispensing, a Canadian review of 5 pertinent studies of automation in outpatients found a significant reduction in the relative risk ratio for identified dispensing errors [7]. A recent UK study conducted in the last quarter of 2019 showed that lookalike-soundalike (LASA) errors represented 25.9% of the total of all *human* dispensing errors [8]. LASA medications is an area where barcode reading by machine would be expected to be potentially error free.

The current literature also provides some indications of how human factors can interact, or fail to interact, with robotics in the dispensing process. During a transitional phase for the introduction of robotics in a community hospital, the average prescription filling time was reduced by 40 seconds per prescription, [9], but the sequencing of technician workflow steps had to be reviewed, and these increased from 17 to 38 seconds, respectively. A more concerning aspect was that workarounds increased from 10% to 36% after the introduction of robotics. We considered this caveat in the present study, particularly in the workflow for prescription to dispensing, and for processes such as inventory and medication labelling. This informed our project plan and, in particular, our plan for integration.

Studies of pharmacy automation generally give an encouraging view of robotics, with the caveat that original pack dispensing via robotic picking can be expected to yield better results in terms of dispensing speed than can unit-dose dispensing. In terms of general automation across the dispensing process, the studies are positive in their reviews of robotic filling of prescriptions and barcode-based medication dispensing, with evidence of reduced error incidence, improved prescription filling time, and completeness of prescriptions.

The Kingdom of Saudi Arabia has been actively engaged in pharmacy automation for a considerable period. There are, however, still *traditional* pharmacies serving communities into which we are introducing automated pharmacies. This gave us an opportunity to make head-to-head comparisons between the 2 systems over an extended period and to gather preimplementation metrics, such as time to filling of prescriptions from the moment a prescription was made or from patient presentation in the case of repeat orders. This addresses a noticeably clear gap in the current literature.

### Objectives

We forecasted growth of our outpatient service at 25% per annum over 5- and 10-year horizons and set out to prepare our outpatient pharmacy service to meet this demand. The overall objective of the study that we conducted alongside our project plan to meet this demand was to establish, using an easily reproducible and reliable methodology, the benefits of an automated and integrated pharmacy dispensing solution versus a traditional outpatient pharmacy system through pre- and postimplementation comparisons. Metrics of FTE freed-up time, the time gained or lost in pharmacy tasks, dispensing error rate, patient satisfaction, and patient wait times were assessed in both comparisons. The study also addressed return on investment (ROI) of automation in the outpatient environment, in terms of productivity and avoidance of error.

The study was undertaken in the northeastern region of Saudi Arabia and was intended to help decision-makers in both the private and public sectors to make more fully informed decisions about the adoption of automation generally and, more specifically, the introduction of outpatient pharmacy automated dispensing systems. The possible intangible benefits of outpatient dispensing automation have not been fully assessed in the scientific literature. These include the opportunity to redeploy highly qualified staff away from routine tasks and to direct them toward more constructive engagement with patients.

## Methods

### Study Design

The study lasted 21 months (September 2018 to June 2020), with a go-live for the automated pharmacy after 11 months (August 2019).

The benefits of an automated and traditional system pre- and postimplementation study is that over the extended period of the study, equally complex patients with diverse issues of infirmity, age, education, pharmaceutical requirements, and health state can be expected to be presented to both systems. A metric of FTE deployment and time gained or lost in pharmacy tasks and in managing each system would therefore be expected to identify how much time for patient counseling and assessment of patient needs is allowed for by each system.

Patient satisfaction in both units was assessed using a standard tool adapted for our facility in a partnership between the pharmacy department and the Press Ganey organization (Textbox 1). The core survey and the questions used have been verified for use in outpatient medical practice [10], and these types of survey are in common use across the United States. The surveys are delivered after each interaction with the outpatient pharmacy via text messaging to smartphones and via email to patients or their carers. The surveys can also be completed on unit-based tablet computers. They are delivered in Arabic and English. Patients are asked to complete a 20-question survey, and although the questions may be altered occasionally for special polling purposes, the core questions related to satisfaction and quality of care, and the 5-point Likert scale (range 1 to 5), remain unchanged, allowing for long-term analysis of trends and assays of the impact of changes in the outpatient pharmacy environment, management, or process on patient satisfaction overall, and for wait time, in particular (ie, three core questions are devoted to this aspect of care).

**Figure 1:**
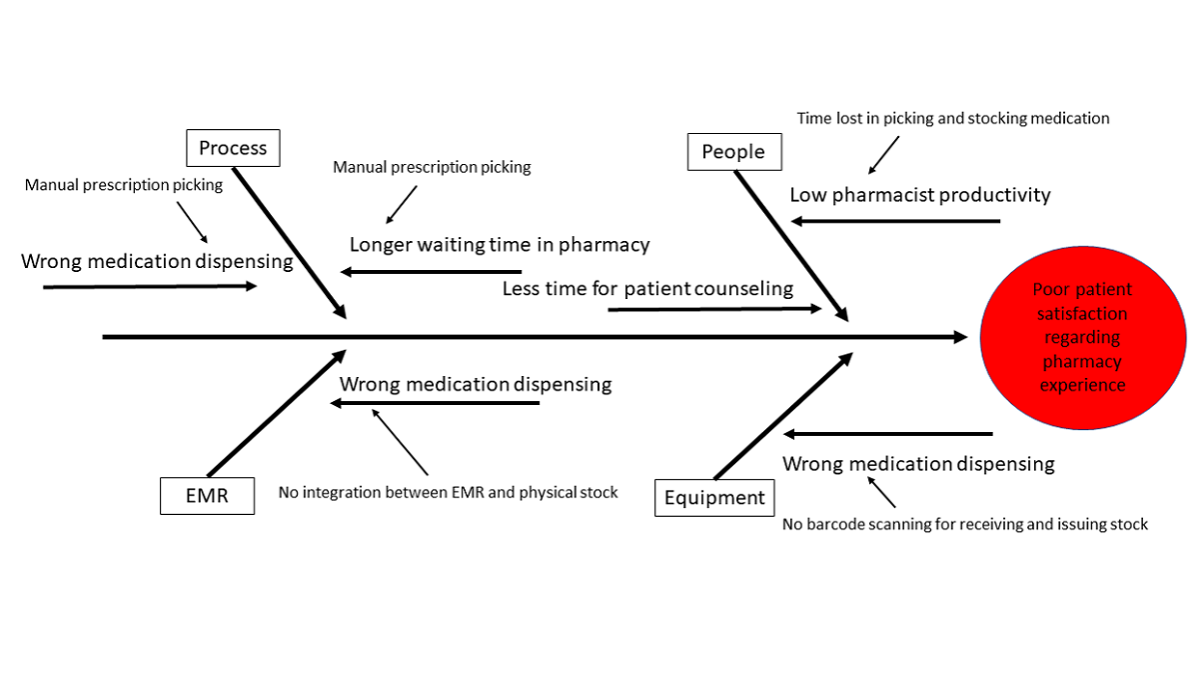
Identified chokepoints and variances in preimplementation processes. EMR: electronic medical record.

Data gathered from the facility awaiting implementation indicated a decline in patient satisfaction regarding pharmacy wait time and a decrease in overall patient satisfaction regarding pharmacy services, which was associated with an increasing average wait time, flat pharmacist productivity, and an increase in reported dispensing errors. This provided benchmarks to measure our impact. It also aided with team selection as we identified process variations and choke points hindering improvement (see Figure 1), and we were able to recruit personnel directly involved at these points into the project team. A review of 1 year of preimplementation data is available in the Results section. Our planned outcome indicator metrics were based on preimplementation data.

**Example questions from the patient satisfaction survey used in both units and pre- and postautomation initiation.**

**For your visit today you were assisted by a staff member.**

**Please answer the following questions with that health care provider in mind.**

(The survey usually takes about two 2 minutes to complete. Some of the core questions are listed below. For each question, the respondent has the following answer options: “very poor,” “poor,” “fair,” “good,” and “very good”)

- Friendliness and courtesy of the care provider.
- Explanations the care provider gave you about your medications and condition.
- Concern the care provider showed for your questions or worries.
- The amount of time the care provider spent with you.
- Degree to which the care provider talked to you using language you could understand.
- The timeliness of the care provider’s interaction with you.
- The time you had to wait to be called or seen by a care provider.
- The time you had to wait before receiving your medications and being able to leave the hospital.

Our initial selection of automation components and systems was guided by a review of the literature. The metrics of technology selection in terms of required storage, picking, and delivery rates was built upon the basics of known pack dispensing rates (2000 articles per day), patient and prescription load per day (1300 patients and 1300 prescriptions), average packs and lines per prescription (10 packs and 10 lines), and lines held (approximately 2000 lines). As noted above, we also forecasted growth of the service at 25% per annum over 5- and 10-year horizons.

The studied outpatient pharmacy operated, at both pre and postimplementation stages, a 24-hour service with peak times between 0900 and 1230 hours and between 1600 and 2200 hours.

Refrigerated items are both stored and dispensed in the department. In terms of storage, we estimated a requirement for 0.67 m^3^ with a capacity of 210 packs per fridge.

Our goal was total automation of the processes of stock management; therefore, we investigated systems with fully automatic input, and this was planned to take place during low patient-volume hours at a minimum rate of 1400 packs/hour input.

We intended to use medication manufacturers’ barcodes without relabeling being undertaken in the input process to the robotic pharmacy unit. Relabeling on input may slow the input by as much as 20%, and there are generally restrictions on the dimensions of packs that can be relabeled at the point of entry into the inventory.

HL7 (the interfacing and standard messaging language for transfer of clinical and administrative data between software applications) capability was required to integrate with our existing health information system (HIS) that supports appointments, medication records, and prescription. The integration of the robot pharmacy unit and these systems was achieved via FutureGate Pharmaflow architecture. The VM-Ware for the robotic suite and interface engine is inside the facility’s firewall, and VPN access is initiated by our facility if access by vendor engineers is required for remote server maintenance.

Rowa Vmax 160 Hardware (Becton, Dickinson and Company) was selected on the basis of the above criteria for picking and input speed and positive integration attributes. Two machines were purchased, each with dimensions of 7 m length × 1.63 m width × 2.5 m height. Each unit has a capacity for 12,500 medications. The architecture involved 10 dispensing desks, with 10 spiral chutes, fed by 2 unidirectional belts with feed gates, serviced by 1 bidirectional belt feeding from four exit points of the 2 robot picking units.

As discussed above, the overall objective of the study running alongside the implementation was to establish, in a reproducible and reliable manner, baseline data to quantify the impact of robotic automation of a centralized outpatient pharmacy system over a period of 10 months. This was part of a system-wide review of the potential further adoption of pharmacy outpatient automation across the organization. These reviews are concerned with value for money, but this goes beyond simple time-saving and efficiency questions and extends into reduction of medication errors and improved patient safety, improved completeness of prescriptions for each dispensing event, shortening patient wait times, and improving the patient’s experience and education level with regard to the medication prescribed.

The pharmacy staffing level in the outpatient department was also roughly equivalent, pre- and postimplementation. See Table 1 for a comparison of the processes in place in pre- and postimplementation.

Process quality indicators and outcome indicators were selected for the study (as described in the Results). These concerned elements pertinent to the process and established criteria, to which we could apply trackers and standards for the implementation and postimplementation periods. These indicators established optimum standards, with ideal values for compliance, with a criterion for each value. Minimum standards were set, as well as transition standards for the implementation and immediate postimplementation periods.

**Table 1:** Pharmacy attributes pre- and postautomation.

Process	Preautomation traditional outpatient pharmacy	Postautomation outpatient pharmacy
Prescribing	CPOE^a^	CPOE
Medication stock-up and record inputting	Manual	Direct loader to robot storage and barcode reading of expiry dates
Space or volume management	Open shelves with secured lock and key for controlled medications. Unknown packs/m^3^	Secured robotic box space calculation and allocation. ~4000 packs/m^3^
Medication picking	Manual	Robotic, barcode multi-picking (8 packages maximum per move)
Dispensing method	Original pack	Original pack.
Dispensed items record keeping	Tracking of each item through HIS^b^	Automatic item deduction from stock level
Delivery to point of care	Manual carry	Conveyor belt and spiral chute
Inventory	Twice per year; manual with HIS reconciliation	Automated storage system maintains consistent inventory

^a^CPOE: computerized provider order entry.

^b^HIS: health information system.

### Study Procedure

The data recorded for analysis were patient anonymized for hospital number, gender, name, date of birth, or other identifiable material. All employees active in the outpatient unit were informed of the data collection taking place.

Becton, Dickinson and Company (BD) Clinical and BD and FutureGate Global Customer Services were engaged to optimize the automated solution, and the BD Medical Affairs department was requested to undertake a deeper analysis of the data. The Medical Affairs department of BD operates as a distinct arm outside of the commercial operations of the company.

### Inclusion and Exclusion Criteria

All formulary items dispensed via the outpatient pharmacy as original pack medications were included in the analysis. Unit-dose medications or blister packs were excluded from the analysis.

## Results

**Figure 2:**
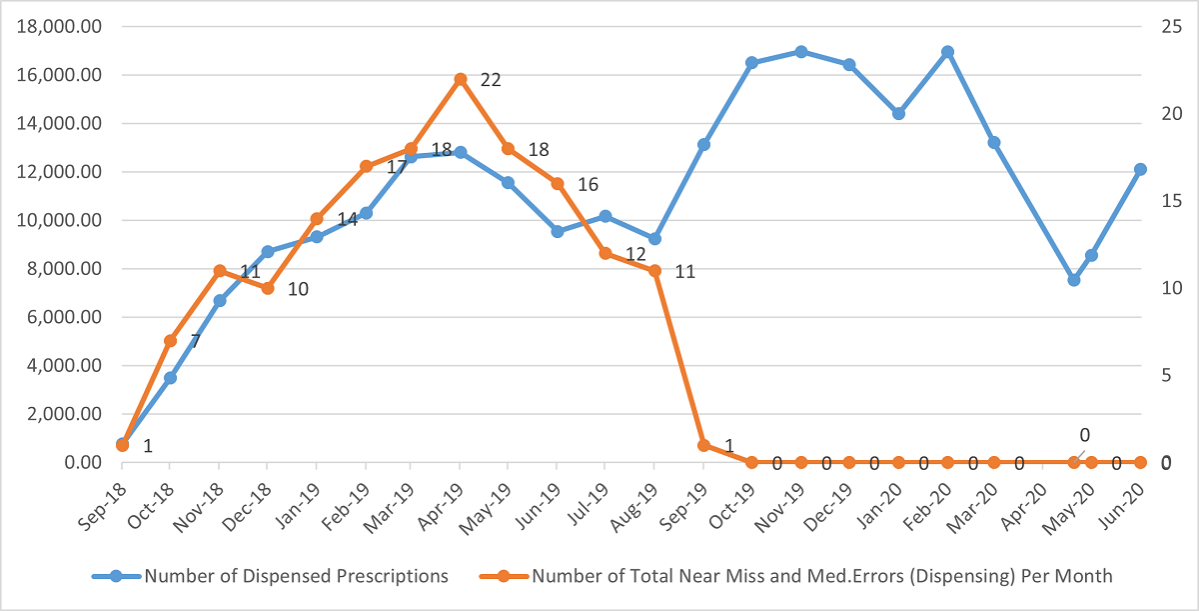
Pre- and postautomation pharmacy total monthly dispensed items versus near-miss and identified medication errors. "Go-Live" August 2019.

During the preimplementation period, the mean number of prescriptions filled per month was 8728.45 (SD 3745.48; minimum 3489; maximum 12,814; median 9544, IQR 2378.75). This value increased during the implementation period to a mean of 13,587.60 (SD 3410.01; minimum 7530; maximum 16,974; median 13,809, IQR 5794), with no change observed for FTE. It was noted that although activity increased significantly in the postimplementation period, the detected error rate also declined rapidly and settled at our target of zero (see Figure 2).

An ongoing review during the implementation of our solution, and of the data aggregated in this period showed that we could start accounting for patient education time (see Tables 2 and 3). This metric had not been gathered in the preimplementation period, as FTEs were constantly focused on picking and dispensing medications and attempting to *keep up* with the patient load. We started to see FTEs taking advantage of the time saved on keep-up tasks, even when this was only 5 minutes per patient, to engage with patients. We placed stretch targets on this time gained of a 30% increase (optimum) with transition targets of 10%-25% increases per patient encounter. How we attempted to guide the activity undertaken with this new *free-time* to increase its benefit, and how we intend to utilize it in the future, for both the pharmacist and the patient is discussed below.

Overall, the study expanded on the findings of the current literature and indicated improved FTE productivity. It also shows the potential for FTE redeployment to more value-added tasks and for further efficiencies.

**Figure 3:**
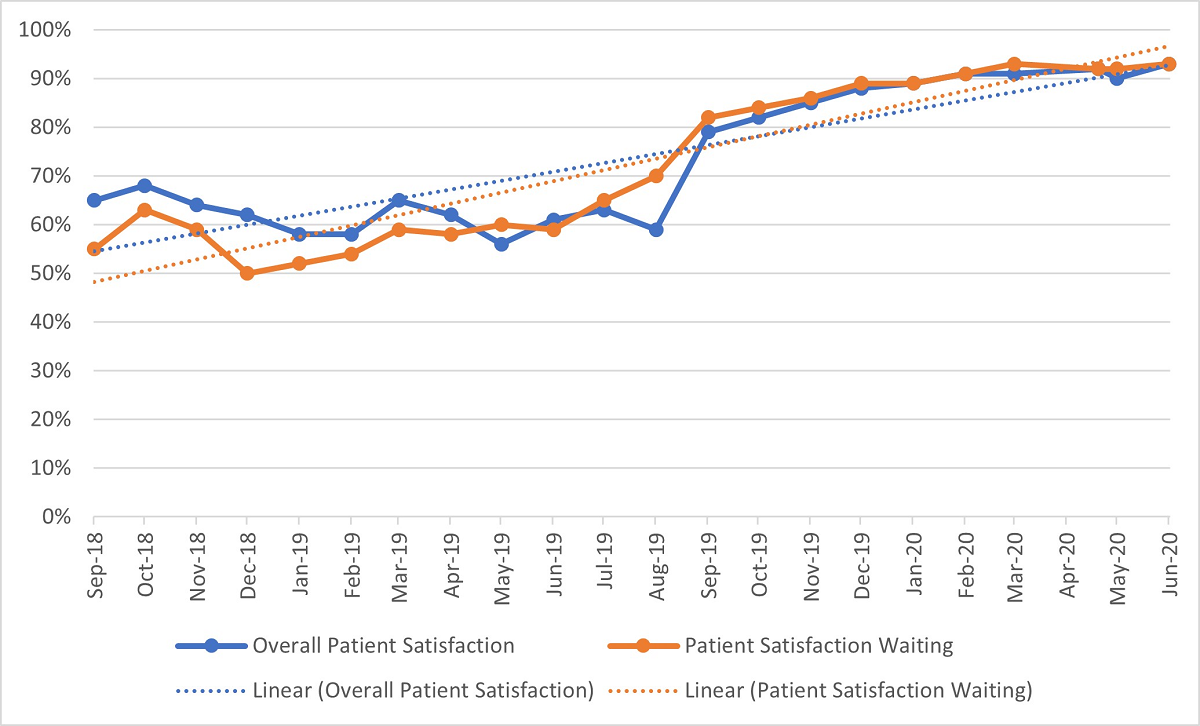
Association between waiting time satisfaction and overall patient satisfaction, automated pharmacy "Go-Live" August 2019.

Overall patient satisfaction was measured pre- and postproject implementation, as it became evident that *freed-up time* was being created by automation for more patient engagement by staff. We wanted to see how much it was valued by patients. Overall patient satisfaction was also clearly and strongly influenced by wait time (see Figure 3).

**Figure 4:**
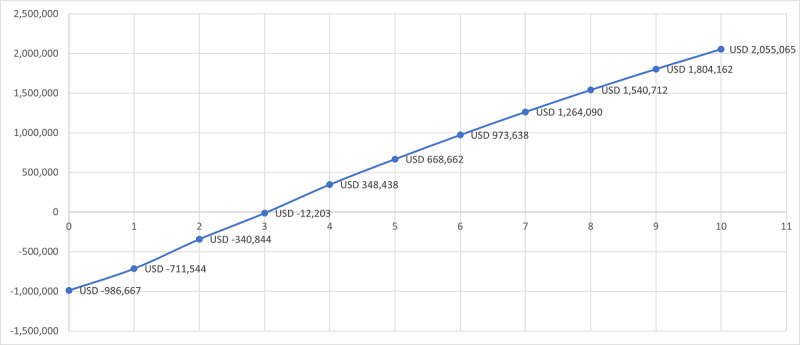
Projected automated-integrated pharmacy return of investment (in USD) with "Tipping Point" at 3.5 years.

The question of discovery during implementation also applied to the question of ROI, which we had not initially set out to measure, but substantial productivity improvements drove us to review this in terms of optimization of manpower, optimization of space utilization, reduction of medication error, cost-savings in terms of improvements in patient safety, avoidance of adverse drug events (ADEs), and reduction in medication loss from expired medications. We were able to ascertain a relatively short-term ROI point of 3.5 years (Figure 4).

**Table 2:** Indicator types and outcomes.

Indicator type and description		Achieved metric	Optimum standard	Minimum standard	Transition standard
**Process quality**					
	Staff education on automated processes	100%	100%	90%	80%
	Staff education on use of freed up time	100%	100%	90%	80%
	Prescriptions filled per month	Meets unit needs	Meets unit needs	Meets unit needs	Meets unit needs
**Outcome**					
	Accuracy of dispensing: error rate per 1000 items dispensed	Zero error	Zero error	Zero error	Zero error
	Patient wait time	53% reduction	50% reduction	45% reduction	35% reduction
	Patient satisfaction specific to wait time	93% overall	>75% overall	15% increase	10% increase
	Pharmacist productivity (daily prescriptions per pharmacist)	33% increase	30% increase	25% increase	10% increase
	Overall patient satisfaction	22% increase, 93% overall	20% increase	15% increase	10% increase
	Patient education time^a^	Future metric (see discussion)	30% increase	25% increase	10% increase

^a^Metric introduced during implementation phase only.

**Table 3:** Pre- and postimplementation metrics.

Indicator type and description		Postimplementation metrics			Preimplementation metrics				
		Mean (SD)	Minimum-maximum	Median (IQR)	Mean (SD)	Minimum-Maximum	Median (IQR)		
**Process quality**									
	Staff education on automated processes	N/A^a^	N/A	N/A	N/A	N/A	N/A		
	Staff education on use of *freed up* time	N/A	N/A	N/A	N/A	N/A	N/A		
	Prescriptions filled per month	13,587.60 (3,410.01)	7530-16,974	13,809 (5794)	8728.45 (3745.48)	(3489-12,814)	9544 (2378.75)		
**Outcome**									
	Accuracy of dispensing: error rate per 1000 items dispensed	0.01 (0.02)	0.00-0.08	0 (0)	1.50 (0.26)	(1.15-2.01)	1.53 (0.35)		
	Patient wait time (min)	7.90 (1.37)	6-11	8.00 (1.5)	15 (5.03)	(5-22)	15.5 (5.5)		
	Patient satisfaction specific to wait time (%)	89 (0.04)	82-93	90 (7)	58.67 (5.60)	50- 70	59 (5)		
	Pharmacist productivity (daily prescriptions per pharmacist)	60 (15)	33-74.4	60.98 (20.25)	43.5 (18.66)	(17.39-63.86)	47.57 (25.19)		
	Overall patient satisfaction (%)	88 (5)	79-93	90 (5.25)	62 (4)	56-68	62 (5.5)		
	Patient education time^b^ (min)	~5	N/A	N/A	N/A	N/A	N/A		

^a^N/A: not applicable.

^b^Metric introduced during implementation phase only.

## Discussion

### Principal Findings

Our overall error rate was lower than those reported in other studies [11-13], and we suggest this is also related to the workflow for stocking and dispensing we utilized. Failures of barcode relabeling (ie, omission of labelling) has been cited as one cause of error in robotic dispensing systems [14]. This potential failure was not observed in our workflow because we do not add barcodes to medications and because we use manufacturer product barcodes at stock input and for picking. The risk of dispensing expired stock noted as a *failure mode* [14] can also be mitigated by using original manufacturer medication container barcodes, thereby removing the step of relabeling that introduces the possibility of mislabeling or omission of this information during input to the robotic unit.

Electronic medical record and CPOE integration allows for forecasting of medication demand, and stock held in the robotic unit and availability in the supply chain has also helped us to mitigate the risk of stockouts that may cause incomplete prescription filling or requiring medication substitution. An automated pharmacy solution cannot exist in isolation—the upstream supply chain is vital, particularly when automated filling is planned.

Arguably, our productivity at 33% per FTE was far greater than that reported in other studies of robotic pharmacies directly serving patients; however, these studies have commonly centered on retail pharmacies, with dispensing patient interfaces completely replacing the dispensing pharmacist [15]. Studies with a greater similarity to ours, as discussed above, are limited in the scientific literature.

In acknowledging the limited literature, it is notable that our results are generally in line with many of the previous findings in this field. Positive *user* satisfaction with a centralized automated-dispensing system with a mean score of 5.52 (SD 1.20; maximum: 7) was reported along with a statistically significant drop in dispensing errors from 2.9% to 1.7% (*P*<.001) in a recent study [11], and a *wrong content* error rate of 0.6%-1.2% recorded in another study [12]. The systems studied were, however, central pharmacies serving diverse inpatient units with automated dispensing cabinets (ADCs) and more traditional ward storage systems, and the ROI estimates given even in the most recent studies are difficult to evaluate against those of the present study, as the system underwent several upgrades over the 8-year study period [11,13]. We were fortunate to have had the same hardware and software from the outset in our automated pharmacy, including the direct loader-to-storage and barcode reading of expiry dates for restocking. An automated pharmacy solution should not be planned or implemented in isolation of its supply needs. We also believe that this automated restocking process was a key reason for the relatively short ROI payoff period of the present study. We may also have benefitted from serving 1 department and 1 community with moderately predictable medication needs and volumes, though with diverse patient subpopulations.

We mapped our dispensing process pre- and postimplementation of the robotic pharmacy (see Figure 1). A similar process-mapping exercise was undertaken in a 2020 French study [13], to more fully uncover the ROI likely to be achieved by implementation of robotic pharmacies. In this study, the FTE costs saved through automation were the most significant gain, followed by stock variation savings [13]. This is entirely similar to our experience, although we arrived at our metrics for the FTE saving through overall productivity per staff count rather than average dispensing time. Our *tipping point* for the ROI at the 3.5-year mark is also similar to that found in this study at 3.75 years [13], and it is comparable to other experiences with medication management automation within facilities (eg, one study on ADCs estimated ROI at 3.8 [minimum: 2.7, maximum: 6.4] years [16]).

### Limitations

We recognize the limitations of this study. No blister packs or unit-dose packs were dispensed, and there was no relabeling or splitting of whole pack medications. This may be an issue for units that wish to split or create custom packs, as this would require new barcode labels for each new patient package, which would increase labor and may slow down operations. This may be an issue with limited prescription fills for high-value medications or if units wish to shorten refill times to increase face-time with patients.

Furthermore, although we instigated education for staff to assist them in effectively utilizing the *free time* gained from automation, it is more difficult perhaps to effectively assay the productivity of this time. For this purpose, in our projected study of the effectiveness of patient education and medication reconciliation processes by pharmacists, we may be able to show a distinct link between increased (and guided) freedom for pharmacists from clerical tasks taken on by automation and improved patient medication adherence.

In this study, an extensive hospital information system was already in place at the time of the switch to automation. Other units without this level of integration between an existing HIS, the CPOE system, and the appointment system may not achieve similar results. However, in non–peer-reviewed regional publications, there have been reports of traditional versus automated head-to-head studies with no HIS present in either scenario that have still shown commendable metrics on improvement in dispensing time and error reduction in outpatient dispensing [17], with a 28.8% increase in complete orders dispensed and a time reduction approaching 96% for mean total prescription filling time for the automated pharmacy. However, the choke point that remained in both systems was from prescription to the initiation of dispensing, which indicates the importance of CPOE integration.

In terms of the hardware deployed, we have not presented a *standard* discount rate for our infrastructure investment (usually for studies of this sort, we would apply amortization over 10-15 years at a 5% discounted annual rate). Nevertheless, this would have brought the ROI tipping point forward from 3.5 years, and current inflationary pressures (excluding pharmaceuticals) are not exacting.

In this study, overall patient satisfaction increased postautomation. We suggest that this outcome is related to the fact that the *freed-up time* created by automation allowed for more patient engagement by staff and because wait time was being reduced. A 2018 survey [18] conducted in an outpatient pharmacy found a strong relationship between overall patient satisfaction and satisfaction with wait time, but we also noted that the most important predictor of patient satisfaction was the quality and quantity of time spent by pharmacists with patients, and how this time was spent to provide information on the dispensed medications and to resolve patient concerns. In general, current levels of satisfaction with this aspect of patient care have been suggested to be less than optimal, with a study on community pharmacies [19] indicating that only 34% of patients were satisfied with the medication counseling they received at their local center, and only 47.3% of surveyed pharmacists were satisfied with the medication counseling they were able to provide. Both patients and pharmacists identified lack of time as a major reason for these subpar outcomes, and both groups were also strongly positive (88% of patients and 73% of pharmacists) about the development of medication counseling standards to guide counseling sessions. As noted in the limitations of our study, although we have gained *free time* for pharmacists in the outpatient department, we cannot be sure of the effective utilization of this time. We have put training in place (see Table 3), but the above consideration of the quality as well quantity of time spent with patients suggests that a more formal and measurable approach to patient counseling is required, if we are to prove the value of creating free time through automation more fully. Focusing on one particular aspect of medication counseling, such as medication regimen adherence, as an outcome key performance indicator would be a logical approach to this issue. It is possible that with increased face-time between pharmacists and patients and a reduction in inconvenience for the patient in each visit to the outpatient pharmacy, there might be an expectation of improved medication adherence and a reduction in ADEs related to incorrect medication usage by patients [20]. Therefore, in follow-up studies, we intend to extend our work to assaying more exactly how this extra time spent by pharmacists with patients affects medication adherence. Nonadherence is a problem of increasing magnitude that particularly affects those with chronic diseases [21] and symptomless conditions [22]. A major concern is that a drop-off of around 50% can be expected during early stages of a regimen, and that this percentage increases over time [23].

The delivery of educational content to patients has been shown to affect adherence rates [24], but this, of course, takes time and utilizes human resources. Our intention is to use a recognized tool, such as the Morisky-Green-Levine Medication Adherence Scale, to gauge outcomes and to confirm any improvement. We believe that increased time with pharmacists will allow patients to increase their knowledge about their disease and treatment and to better understand their own psychological needs related to regimens. This will lead to improved adherence scores. We also believe that reducing wait times may improve adherence through allowing for smaller prescription fill volumes and more frequent outpatient department visits and, therefore, increased contact time with pharmacists. Pharmacists play a major role in health promotion activities and in providing health education for patients, particularly around their medication regimen [25]. Automation may be the key to freeing them from non–value-added tasks for this vital undertaking, but any automation solution must also be seamlessly and completely integrated into the facility’s appointments, medication records, and prescription software systems for this to be achievable.

### Conclusions

The robotic pharmacy solution studied was highly effective, but a robust upstream supply chain is vital to ensure adequate stock levels, particularly when automated filling is planned. The automation solution must also be seamlessly and completely integrated into the facility’s software systems for appointments, medication records, and prescription in order to garner its full benefits.

Overall patient satisfaction with pharmacy services is strongly influenced by wait time, and follow-up studies are required to identify how to use this positive effect and how to make optimal use of the *freed-up* pharmacist time. The extra time spent by pharmacists with patients and the opportunity for complete overview of the patient’s medication history that full integration provides, may allow us to address challenging issues such as medication nonadherence. Reduced wait times may also allow for smaller prescription fill volumes and more frequent outpatient department visits, thereby allowing patients to have increased contact time with pharmacists.

## Abbreviations

ADC: automated dispensing cabinet
ADE: adverse drug event
BD: Becton, Dickinson and Company
CPOE: computerized provider order entry
DDS: drug distribution system
FTE: full-time equivalent
HIS: health information system
LASA: lookalike-soundalike
ROI: return of investment

## References

[1] Hynniman C (1967). Lamy P; Outpatient Pharmacy Automation. American Journal of Hospital Pharmacy.

[2] Sng Y, Ong CK, Lai YF (2018). Approaches to outpatient pharmacy automation: a systematic review. Eur J Hosp Pharm.

[3] Ahtiainen HK, Kallio MM, Airaksinen M, Holmström A (2019). Safety, time and cost evaluation of automated and semi-automated drug distribution systems in hospitals: a systematic review. Eur J Hosp Pharm.

[4] Busquets FB, Celma MS, Escoda AC, Suárez JA, Comas AM, Moreno MC, Cayuela MR, Foguet JC, Riba RF (2016). DD-025 Automatic storage system: Impact in reducing medication errors in a paediatric hospital. Eur J Hosp Pharm.

[5] Tam V, Lim M (1997). Patients' Perceptions and Expectations of Outpatient Pharmacy Services in a Teaching Hospital. Int J Pharm Prac.

[6] Tan W, Chua S, Yong K, Wu T (2009). Impact of pharmacy automation on patient waiting time: an application of computer simulation. Ann Acad Med Singap.

[7] Canadian Agency for Drugs and Technologies in Health (2010). Automated Medication Dispensing Systems: A Review of the Clinical Benefits, Harms, and Cost-Effectiveness. Automated Medication Dispensing Systems: A Review of the Clinical Benefits, Harms, and Cost-Effectiveness.

[8] National Pharmacy Association (2019). NPA medication safety update (MSO report). NPA medication safety update (MSO report) Quarter 4 2019 (England).

[9] Walsh K, Chui M, Kieser M, Williams S, Sutter S, Sutter J (2011). Exploring the impact of an automated prescription-filling device on community pharmacy technician workflow. J Am Pharm Assoc (2003).

[10] Presson AP, Zhang C, Abtahi AM, Kean J, Hung M, Tyser AR (2017). Psychometric properties of the Press Ganey® Outpatient Medical Practice Survey. Health Qual Life Outcomes.

[11] Berdot Sarah, Korb-Savoldelli Virginie, Jaccoulet Emmanuel, Zaugg Vincent, Prognon Patrice, Lê Laetitia Minh Maï, Sabatier Brigitte (2019). A centralized automated-dispensing system in a French teaching hospital: return on investment and quality improvement. Int J Qual Health Care.

[12] Franklin D, O'Grady K, Voncina L, Popoola J, Jacklin A (2008). An evaluation of two automated dispensing machines in UK hospital pharmacy International Journal of Pharmacy Practice, 16. International Journal of Pharmacy Practice.

[13] Mathy C, Pascal C, Fizesan M, Boin C, Délèze N, Aujoulat O (2020). Automated hospital pharmacy supply chain and the evaluation of organisational impacts and costs. Supply Chain Forum: An International Journal.

[14] Rodriguez-Gonzalez C, Herranz-Alonso A, Escudero-Vilaplana V, Ais-Larisgoitia M, Ribed-Sanchez A, Tovar-Pozo M, Sanjurjo-Saez M (2016). A risk analysis method to evaluate the impact of robotic dispensing on patient safety. Eur J Hosp Pharm.

[15] Ruhle F, Braun R, Ostermann H (2009). Impact of robotic dispensing machines in german pharmacies on business performance indicators. Libyan J Med.

[16] Bonnabry P, François Olivia (2020). Return on investment: a practical calculation tool to convince your institution. Eur J Hosp Pharm.

[17] Waterson J, Al-Naqby A, El-Shamy A (2019). UAE Ministry of Health: Leading on automation in outpatient medication dispensing. Omnia Health.

[18] Martínez-López-de-Castro Noemí, Álvarez-Payero Miriam, Martín-Vila Alicia, Samartín-Ucha Marisol, Iglesias-Neiro P, Gayoso-Rey M, Feijoo-Meléndez Débora, Casanova-Martínez Cristina, Fariña-Conde Miguel, Piñeiro-Corrales Guadalupe (2018). Factors associated with patient satisfaction in an outpatient hospital pharmacy. Eur J Hosp Pharm.

[19] Yang S, Kim D, Choi HJ, Chang MJ (2016). A comparison of patients' and pharmacists' satisfaction with medication counseling provided by community pharmacies: a cross-sectional survey. BMC Health Serv Res.

[20] Oñatibia-Astibia Ainhoa, Malet-Larrea Amaia, Larrañaga Belen, Calvo B, Ramírez Dulce, Cantero I, Goyenechea E, Gastelurrutia, Garay (2019). Tailored interventions by community pharmacists and general practitioners improve adherence to statins in a Spanish randomized controlled trial. Health Serv Res.

[21] World Health Organization (2003). Adherence to Long-Term Therapies. Evidence for Action.

[22] Van Driel M, Morledge M, Ulep R, Shaffer J, Davies P, Deichmann R (2016). Interventions to Improve Adherence to Lipid-Lowering Medication. Cochrane Database Syst Rev.

[23] Huser MA, Evans TS, Berger V (2005). Medication adherence trends with statins. Adv Therapy.

[24] Gujral G, Winckel K, Nissen LM, Cottrell WN (2014). Impact of community pharmacist intervention discussing patients' beliefs to improve medication adherence. Int J Clin Pharm.

[25] Beshir SA, Bt Hamzah NH (2014). Health promotion and health education: perception, barriers and standard of practices of community pharmacists. International Journal of Health Promotion and Education.

